# Investigation of Tick Species and Seasonal Population Dynamics in Sheep, Cattle, and Goats in Ağrı Province, Türkiye

**DOI:** 10.3390/pathogens15050547

**Published:** 2026-05-19

**Authors:** Ali Bilgin Yilmaz, Milad Afsar, Muhammed Yasul, Muhammed Ahmed Selcuk, Mahsa Torkamanian-Afshar, Asım Ozbek, Hasan Yilmaz

**Affiliations:** 1 Faculty of Health Science, Van Yuzuncu Yil University, 65000 Van, Türkiye; 2 Department of Parasitology, Faculty of Medicine, Van Yuzuncu Yil University, 65000 Van, Türkiye; mtamilad@gmail.com (M.A.); hasanyilmaz@yyu.edu.tr (H.Y.); 3 Faculty of Health Sciences, Iğdır University, 76000 Iğdır, Türkiye; m.yasul@igdir.edu.tr; 4 Department of Parasitology, Faculty of Veterinary Medicine, Siirt University, 56000 Siirt, Türkiye; ahmed.selcuk@siirt.edu.tr; 5 Software Engineering Department, Engineering and Natural Sciences Faculty, Istanbul Topkapi University, 34087 İstanbul, Türkiye; mahsatorkamanianafshar@topkapi.edu.tr; 6 Medical Laboratory Techniques Program, Health Services Vocational School, Siirt University, 56100 Siirt, Türkiye; asimozbek@siirt.edu.tr

**Keywords:** livestock health, prevalence, seasonal dynamics, Türkiye, vector-borne diseases

## Abstract

This study examined the distribution and seasonal population dynamics of tick species infesting cattle, sheep, and goats in Ağrı Province, Türkiye. From January to December 2024, 913 hosts were examined, and 545 intact tick samples were collected from 386 infested animals and morphologically identified. The overall sex ratio was 52.7% female and 47.3% male. The identified species included *Dermacentor niveus*, *D. marginatus*, *Rhipicephalus sanguineus*, *R. turanicus*, *R. bursa*, *Haemaphysalis punctata*, *Hae. sulcata*, *Hyalomma marginatum*, *H. anatolicum anatolicum*, *H. anatolicum excavatum*, and *H. detritum detritum*. Seasonal tick activity peaked in summer (51.7%) and spring (42.9%), with a significant decline in autumn (χ^2^ = 48.72, df = 3, *p* < 0.001). No active ticks were observed in winter due to the harsh continental climate. Adult *Hyalomma* ticks, which are known potential vectors of Crimean–Congo hemorrhagic fever (CCHF), remained active except during winter. The high prevalence of these vector species suggests a need for further investigation into pathogen circulation in the region. This research provides a scientific foundation for integrated vector control and surveillance programs within the One Health framework.

## 1. Introduction

Türkiye possesses a rich tick fauna due to its geographical location and climatic diversity. Recent studies continue to provide new data for the comprehensive determination of tick diversity in both domestic animals and wildlife [1,2]. Ticks (Ixodida) are recognized as the second most important group of vectors for human and animal health worldwide, after mosquitoes, due to their vital role in transmitting many pathogens to humans and animals [3]. According to the updated taxonomic list published as of March 2025, there are 786 valid species of Ixodidae (hard ticks) described globally [4]. Accurate and reliable identification of these species is essential for effective public health surveillance and the control of tick-borne diseases [5].

Because of its location along an intercontinental transit corridor and its role as a hub for important migratory bird routes, Türkiye has favorable ecological conditions that support the presence of medically important tick genera such as *Hyalomma*, *Ixodes*, *Dermacentor*, and *Rhipicephalus*. In particular, Crimean–Congo Hemorrhagic Fever (CCHF), which has become a significant public health concern since 2004, and its primary vectors, *Hyalomma* species (which account for 46.99% of human tick bites), are the main focus of national tick surveillance efforts [2]. Despite extensive research, the lack of standardized methods in tick studies and the limitations of species-level diagnostics remain major research gaps. In the absence of direct pathogen screening, monitoring vector distribution and seasonal activity serves as a preliminary indicator for assessing the potential risk of tick-borne diseases [6].

Ağrı Province, located in the Eastern Anatolia Region at an average altitude of 1640 m, provides a highly suitable environment for tick populations to thrive due to its distinct continental climate and a high pasture-to-meadow ratio of 36%, roughly 2.5 times the national average. According to 2024 Türkiye Statistical Institute (TÜİK) data (2024) [7], the province accounts for 2.2% of Türkiye’s total cattle population, making it a key area for research on tick–host interactions and vector-borne disease risks. This study contributes a significant dataset to the regional literature by detailing the district-level spatial distribution and seasonal dynamics that have not been previously examined in the province of Ağrı.

This study aims to identify tick specimens collected from cattle, sheep, and goats in Ağrı province through microscopic examination and standardized taxonomic criteria. Unlike previous research, this work offers district-level spatial analysis with statistical significance testing and detailed seasonal dynamics using chi-square analysis with Bonferroni correction. The results are expected to clarify how the region’s ecological and climatic conditions affect tick population structure and behavior, thereby providing a solid scientific basis for local surveillance efforts. Additionally, heavy tick infestation in livestock is a key factor increasing the risk of zoonotic disease transmission, such as Crimean–Congo Hemorrhagic Fever, which is endemic in humans in the region, thereby reinforcing the importance of the One Health approach.

## 2. Materials and Methods

A total of 913 animals cattle (*Bos taurus*), sheep (*Ovis aries*), and goats (*Capra hircus*) were examined in the study. Animals were selected using a simple random sampling method from 20 different farms across the eight districts of Ağrı Province (2–4 farms per district, depending on local livestock density). To ensure the reliability of the infestation data and avoid potential confounding factors, only animals that had not been treated with any acaricides or anti-tick medications within the 30 days prior to examination were included. The sampling process was conducted during routine field visits by veterinarians at monthly intervals from January to December 2024. To prevent sampling bias, different animal herds were screened during each visit, and repeated examinations of the same individual animals were avoided. To ensure comparability of seasonal data, a consistent sampling effort was maintained across all seasons (approximately 228 animals examined per season). A total of 913 animals were examined throughout the study period.

During tick collection, the entire body surface of each animal (especially the inner ear, neck, axilla, inguinal region, and under the tail) was systematically examined. Ticks were manually removed with fine-tipped forceps to preserve their biological integrity and stored in tubes containing 70% ethanol. All active life stages (larvae, nymphs, and adults) were included; however, to ensure taxonomic accuracy, morphological analyses focused on adults. A total of 21 ticks were collected from goats; however, 14 were excluded from species-level analysis due to physical damage. This damage occurred primarily during manual removal or as a result of long-term preservation in 70% ethanol, leading to the loss of diagnostic structures such as the capitulum or adanal shields. Despite these limitations, damaged specimens were identified at the genus level as *Rhipicephalus* spp. (n = 9) and *Hyalomma* spp. (n = 5), consisting mainly of adults and nymphs. The collected samples were transported to the Parasitology Research Laboratory at Van Yüzüncü Yıl University Faculty of Medicine. Ticks were examined under a stereomicroscope (SOIF ST8050-B8LS, Shanghai, China) at 10×–40× magnification; identifications were confirmed by two independent experts. 

### Statistical Analysis

The data collected in this study were analyzed using IBM SPSS Statistics (version 26.0) and MINITAB (version 19.0). Categorical variables, such as infestation status, host species, district, and season, were summarized with frequency distributions and percentages.

The primary outcome variable was tick infestation status. Differences in infestation prevalence among host species, districts, and seasons were assessed using the Pearson chi-square (χ^2^) test. When the overall chi-square test indicated a statistically significant difference (*p* < 0.05), pairwise comparisons were conducted using the two-proportion Z-test with Bonferroni correction to control for Type I error from multiple comparisons. The number of pairwise comparisons (k) and the corresponding Bonferroni-adjusted significance levels (α_adjusted = 0.05/k) were calculated separately for each analysis: For district-level comparisons, each district was compared against the overall provincial average (8 comparisons); therefore, the Bonferroni-adjusted significance level was set at α = 0.05/8 = 0.00625. For host species comparisons, each species was compared against the overall average (3 comparisons); adjusted α = 0.05/3 = 0.0167. For seasonal comparisons, each season was compared against the overall average (4 comparisons); adjusted α = 0.05/4 = 0.0125. A *p*-value of less than 0.05 was considered statistically significant unless noted otherwise.

A *p*-value of less than 0.05 was considered statistically significant unless noted otherwise. In the study, two fundamental parameters were used to evaluate epidemiological data: Prevalence was calculated as the ratio of the number of animals infected with ticks to the total number of animals examined, and Tick Load was calculated as the average number of ticks per infected animal. Due to non-normal distribution (tested by Shapiro–Wilk, *p* < 0.05), the non-parametric Kruskal–Wallis test was used to compare tick loads across host species.

Infection prevalence was calculated as the ratio of the number of animals carrying ticks to the total number of animals examined. In field studies, the presence of tick specimens unsuitable for morphological identification (damaged or fragmented) was also recorded as ‘positive infection’; this prevented specimens that could not be identified to species level from masking the prevalence data.

## 3. Results

### 3.1. District-Level Distribution of Tick Infestation

A total of 913 animals were examined across eight districts of Ağrı Province. The overall tick infestation rate was 42.3% (386/913). District-level infestation rates ranged from 29.7% (Eleşkirt) to 52.5% (Hamur). The chi-square test showed a statistically significant difference in infestation rates among districts (χ^2^ = 24.31, df = 7, *p* = 0.001).

**Figure 1:**
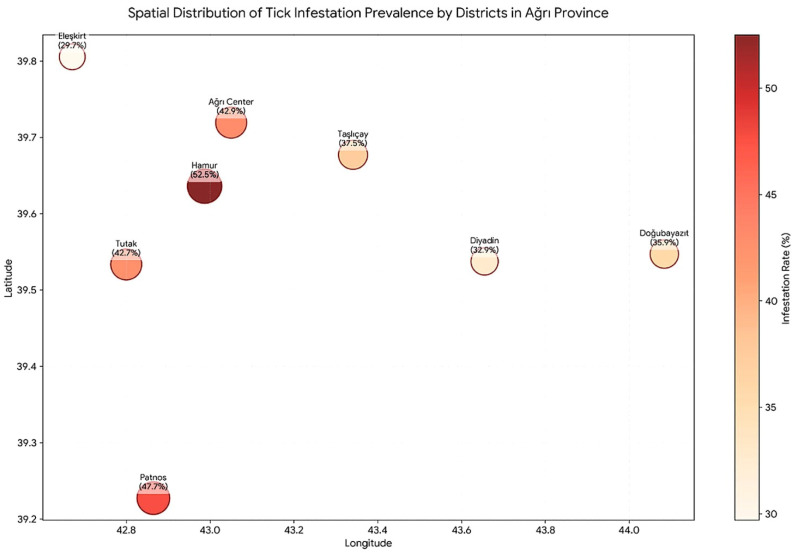
A color-coded map displays infestation rates for Ağrı province districts: Eleşkirt 29.7%, Diyadin 32.9%, Doğubayazıt 35.9%, Taşlıçay 37.5%, Ağrı Center 42.9%, Tutak 42.7%, Patnos 47.7%, Hamur 52.5%.

Pairwise comparisons using the two-proportion Z-test with Bonferroni correction (adjusted α = 0.05/8 = 0.00625) showed that Eleşkirt (29.7%) and Diyadin (32.9%) had significantly lower infestation rates compared to the provincial average (*p* < 0.001 for both). No significant differences were found between Hamur (52.5%) and the provincial average (*p* = 0.521), nor between Patnos (47.7%) and the provincial average (*p* = 0.226) after correction.Taşlıçay (37.5%) showed a *p*-value of 0.006, which is statistically significant after Bonferroni correction (adjusted α = 0.00625; *p* = 0.006 < 0.00625). In comparisons between districts, tick prevalence in Eleşkirt and Diyadin districts was significantly lower than in the other study areas (p<0.05) (Table 1, Figure 1).

#### 3.1.1. Host Species and Infestation Rates

A clear hierarchy was observed among host species in terms of infestation rates. Our findings reveal that cattle and sheep are much more susceptible to tick infestation compared to goats. The low level of infestation observed in sheep can be explained by the physical barrier effect of wool structure on tick attachment [8]. The lower prevalence observed in goats in this study may be linked to their relatively smaller numbers in the sample or potential sampling limitations, despite their natural foraging habits in shrubby and rugged areas that typically offer ideal microhabitats for ticks [9]. When the distribution of the total tick collection was examined, cattle were found to be the dominant host group. This situation is consistent with the large body surface area of cattle and the biological preference of *Hyalomma* ticks, which are dominant in the region, for large ruminants [9,10]. While goats showed a lower prevalence of infestation in this study, the observed differences in tick load (mean intensity) between goats and cattle should be interpreted with caution. The exclusion of severely damaged specimens from the taxonomic analysis likely resulted in an underestimation of the actual tick burden in goats [6]. Furthermore, as a formal statistical comparison of mean intensity was not performed, these observations should be considered as preliminary trends that require further investigation with larger, intact sample sizes to accurately assess host preferences [9].

To provide a standardized comparison, tick load was analyzed as the mean intensity (the average number of ticks per infested animal). Cattle exhibited the highest tick load (1.20 ± 1.45 ticks/animal), which was significantly greater than that of sheep and goats (*p* < 0.05). This reflects both host ecology and a larger body surface area, which facilitates higher tick attachment rates (Table 2).

#### 3.1.2. Tick Species Composition

A total of 545 morphologically intact tick specimens were examined for taxonomic identification and host distribution. Host-based analysis showed that most ticks were collected from cattle (65.5%), followed by sheep (33.2%), and goats (1.3%). Because some of the samples collected from goats were damaged during transport and storage (alcohol concentration), species identification for these animals was performed only on the remaining intact samples (n = 7); however, all animals were included in the calculation of infestation prevalence. The overall sex distribution of the identified ticks was slightly female-biased, with 52.7% (n = 287) females and 47.3% (n = 258) males.

Taxonomic analysis identified 11 tick species. The most common species, in order, were: *Hyalomma anatolicum anatolicum* (20.55%), *Hyalomma marginatum* (15.22%), *Rhipicephalus turanicus* (13.21%), *Hyalomma anatolicum excavatum* (12.84%), and *Rhipicephalus bursa* (11.92%). Less frequently detected species included *Haemaphysalis sulcata* (7.52%), *Haemaphysalis punctata* (6.25%), *Rhipicephalus sanguineus* (4.77%), *Dermacentor marginatus* (3.48%), *Hyalomma detritum detritum* (2.56%), and *Dermacentor niveus* (1.65%). At the genus level, *Hyalomma* accounted for 51.17% of the total, making it the dominant genus, followed by *Rhipicephalus* (29.9%) and *Haemaphysalis* (13.77%) (Table 3).

#### 3.1.3. Seasonal Distribution of Tick Activity

In this study, ‘tick activity’ refers to the seasonal prevalence and density of ticks collected from the examined hosts. Seasons were explicitly defined as follows: Winter (December–February), Spring (March–May), Summer (June–August), and Autumn (September–November). Tick activity exhibited distinct seasonal variation (χ^2^ = 48.72, df = 3, *p* < 0.001). Despite maintaining a consistent sampling effort during the winter months (with an equal number of animals examined as in other seasons), no active tick infestation was encountered. This total absence of ticks in winter is attributed to the extreme continental climate of Ağrı province, characterized by its high altitude (approx. 1640 m), prolonged snow cover, and sub-zero temperatures that often drop below −20 °C, effectively forcing ticks into diapause and inhibiting their host-seeking activity [11]. The activity cycle on hosts resumed in spring, peaking during the summer months.

Pairwise comparisons confirmed that spring and summer had significantly higher tick activity than autumn (*p* < 0.001 for both), with no statistically significant difference between spring and summer (*p* = 0.062).

Species-specific analyses showed that *H. anatolicum anatolicum* and *H. marginatum* peaked in summer with 65 and 59 specimens, respectively. In contrast, *D. niveus* and *D. marginatus* were mainly active in spring, while *R. turanicus* and *R. bursa* remained active in both spring and summer (Table 4).

## 4. Discussion

This study provides a detailed analysis of the epidemiological features and seasonal activity patterns of ixodid tick populations in Ağrı province, a high-altitude (1640 m) and strategically important livestock production hub in Eastern Anatolia. The overall infestation rate of 42.3% clearly indicates that traditional pasture-based animal husbandry practices in the region significantly increase tick-host interactions. This finding aligns with prevalence rates reported from neighboring provinces such as Iğdır, 33.7–54.9%, and Van, 61.7–70.8% [12], while also underscoring notable regional differences compared to the much lower prevalence rates recorded in western Türkiye, such as Afyonkarahisar, 18.1–34.8% [13]. This spatial variation further supports the idea that the unique biogeographical landscape and microclimate of Ağrı province create ideal conditions for tick survival and growth.

### 4.1. Spatial Distribution of Tick Infestation

Spatial analyses conducted at the district level revealed a statistically significant uneven distribution of tick infestation across the study area (overall χ^2^ = 24.31, df = 7, *p* = 0.001). This supports ecological principles, indicating that tick populations in the family Ixodidae are highly sensitive to habitat features, host density, and microclimatic factors, leading to localized clusters in the field [14]. Although the highest proportional infestation rate was recorded in Hamur District (52.5%), pairwise comparisons with Bonferroni correction showed that this rate was not significantly different from the overall provincial average (*p* = 0.521), suggesting that tick-host interactions maintain a relative balance in this district. In contrast, the significantly lower prevalence observed in the Eleşkirt (29.7%) and Diyadin (32.9%) districts, compared to the provincial average (*p* < 0.001 for both), suggests that district-specific environmental or management factors may constrain local tick populations. Taşlıçay (37.5%) had a *p*-value of 0.006, which was statistically significant after Bonferroni correction (adjusted α = 0.00625), indicating that its infestation rate is significantly lower than the provincial average. These spatial differences might be influenced by environmental factors such as altitude, vegetation diversity, grazing behavior, and microclimate. Although these variables were not directly measured in the present study, they are recognized as potential drivers of tick population dynamics and host-seeking efficiency in similar agro-ecological zones [10]. Therefore, conducting a detailed study of local factors affecting tick distribution is essential for regional risk assessment and the development of targeted control strategies.

### 4.2. Host Species and Infestation Patterns

A clear hierarchy was observed among host species in terms of infestation rates. Our findings reveal that cattle and sheep are much more susceptible to tick infestation compared to goats. This low level of infestation observed in sheep can be explained by the physical barrier effect of wool structure on tick attachment [9].

The lower prevalence observed in goats in this study may be linked to their relatively smaller numbers in the sample or potential sampling limitations, despite their natural foraging habits in shrubby and rugged areas that typically offer ideal microhabitats for ticks. When the distribution of the total tick collection was examined, cattle were found to be the dominant host group. This situation is consistent with the large body surface area of cattle and the biological preference of *Hyalomma* ticks, which are dominant in the region, for large ruminants [9,10]. While goats showed a lower prevalence of infestation in this study, the observed differences in tick load (mean intensity) between goats and cattle should be interpreted with caution. The exclusion of severely damaged specimens from the taxonomic analysis likely resulted in an underestimation of the actual tick burden in goats [15]. Furthermore, as a formal statistical comparison of mean intensity was not performed, these observations should be considered as preliminary trends that require further investigation with larger, intact sample sizes to accurately assess host preferences [9].

Among the 786 valid Ixodidae species listed in the foundational taxonomic reference by Guglielmone et al. (2010) [16] and subsequent revisions, the genus *Hyalomma* is an important group that warrants careful public health monitoring due to its high vector competence [17]. The dominant presence of the genus *Hyalomma* (51.1%) among the 11 tick species identified in this study aligns with current systematic data on the tick fauna of Türkiye [18]. Of the documented tick species, *H. a. anatolicum* (20.55%) and *H. marginatum* (15.22%) were the most common. Notably, both species are widely recognized in the literature as the main vectors of CCHF virus [19].

Although this study did not include direct pathogen testing, the high abundance and strong affinity of these capable vectors serve as important epidemiological indicators of potential vector-borne disease risk in the region [2]. The absence of certain widespread species commonly reported in other studies may be due to the unique ecological conditions of Ağrı province, including its high altitude and continental climate, as well as the focus on ruminant hosts in our sampling approach [20]. The tick species identified on domestic ruminants in the Ağrı region are congruent with the general tick fauna of Türkiye. However, recent research on wildlife across the country demonstrates that tick biodiversity is being further enriched with new species records, such as *Ixodes trianguliceps*, moving beyond the scope of studies focused solely on domesticated animals [1].

### 4.3. Seasonal Dynamics and Ecological Adaptations

The seasonal patterns of tick populations indicate that their biological activities are highly responsive to environmental temperature thresholds [21]. Pairwise tests confirmed that spring and summer had significantly higher tick activity than autumn (*p* < 0.001 for both), while there was no significant difference between spring and summer (*p* = 0.062). Tick population dynamics followed a distinct seasonal cycle. The peak of activity in spring and summer indicates that temperature and humidity conditions in the region reach optimal levels for tick development [20,22]. The sharp decline in autumn and the absence of ticks in winter demonstrate that tick presence on hosts is directly dependent on seasonal temperature variations [9,23].

The marked increase in activity during spring and summer is closely related to heightened metabolic and behavioral activity in thermophilic *Hyalomma* species as a response to rising temperatures [12]. Conversely, the complete halt of activity during winter months is directly linked to the region’s harsh continental climate, a phenomenon explained by diapause mechanisms that enable ticks to survive extreme cold [21]. Additionally, the emergence of *Dermacentor* species, particularly *D. marginatus,* during cooler periods such as spring demonstrates that this genus can maintain host-seeking behavior even at lower temperatures, supporting the “cold-season tick” concept described in the literature [14]. Species-specific analyses showed that *H. anatolicum anatolicum* and *H. marginatum* peaked during summer with 65 and 59 specimens, respectively, while *D. niveus* and *D. marginatus* were primarily active in spring. In contrast, *R. turanicus* and *R. bursa* remained active during both spring and summer, reflecting their broader ecological tolerance.

Study Limitations

Despite the significant findings regarding tick distribution in Ağrı, several limitations of this study must be acknowledged. First, tick identification was based solely on morphological characteristics; although standard taxonomic keys were used, molecular techniques could provide higher resolution for distinguishing closely related species or identifying damaged specimens. Second, the exclusion of severely damaged ticks, particularly those collected from goats, may have led to an underestimation of the tick burden and species diversity for certain host groups. Third, this study did not include pathogen detection (CCHF or Borrelia) within the collected ticks, which limits our ability to provide a complete picture of the zoonotic risk beyond vector presence. Finally, while sampling effort was kept consistent across seasons, environmental factors and changes in host availability could still introduce subtle sampling biases. Future studies incorporating molecular identification and longitudinal pathogen screening are necessary to build upon these foundational results.

## 5. Conclusions

In conclusion, the overall tick prevalence of 42.3% identified in Ağrı province and the widespread dominance of *Hyalomma* species, recognized as the main vectors of Crimean-Congo Hemorrhagic Fever virus, highlight the region’s highly dynamic vector ecology. The pronounced spatial heterogeneity in infestation rates across districts and the significant variations observed among host species underscore the complexity of tick ecology in the region. These findings emphasize that vector management strategies should be localized and host-specific, targeting the unique infestation patterns of different livestock species and district-level environmental foci.

Although the current study did not include direct pathogen screening, the notable presence of tick species with high vector capacity is a critical indicator of potential epidemiological risk to public health and livestock management, necessitating further investigations into the local pathogen landscape. These findings emphasize the need for targeted and proactive tick control strategies that account for host species preferences, localized microfoci, and species-specific seasonal activity patterns, rather than relying solely on traditional, generalized methods. Integrated vector control programs based on the One Health approach are expected to form a strategic foundation for managing future zoonotic threats and reducing losses in animal production. This research provides essential data to strengthen regional surveillance infrastructure and guide future epidemiological studies, including pathogen screening and acaricide resistance monitoring.

## Figures and Tables

**Table 1:** Distribution of tick infestation rates in Ağrı province and its districts.

District	Animals Inspected (n)	Animals with Ticks (n)	Infestation Rate (%)	*p*-Value *
Ağrı Centrum	84	36	42.9	0.061
Diyadin	76	25	32.9	<0.001 †
Doğubayazıt	103	37	35.9	<0.001 †
Eleşkirt	91	27	29.7	<0.001 †
Hamur	78	41	52.5	0.521
Patnos	350	167	47.7	0.226
Taşlıçay	56	21	37.5	0.006
Tutak	75	32	42.7	0.069
Total	913	386	42.3	0.001

Pairwise comparisons versus overall provincial average using two-proportion Z-test with Bonferroni correction (adjusted α = 0.00625) * † Statistically significant after correction.

**Table 2:** Distribution of ticks infestation rates in sheep, cattle, and goats based only on animals with intact tick specimens suitable for morphological identification.

Host Species	Animals Inspected (n)	Animals with Ticks (n)	Infestation Rate (%)	*p*-Value *
Cattle	478	181	37.9	0.008 †
Sheep	297	137	46.1	0.810
Goat	138	21	15.2	0.006 †
Total	913	386 ‡	42.3	0.002

Pairwise comparisons versus overall provincial average using two-proportion Z-test with Bonferroni correction (adjusted α = 0.0167). Overall χ^2^ = 12.64, df = 2, *p* = 0.002.* † Statistically significant after correction. ‡ A total of 386 animals were infested (see Table 1). However, 47 animals carried only damaged or fragmented tick specimens that were not suitable for morphological species identification. These 47 animals are included in Table 1 but excluded from this table. Statistical comparsions in this table are based on the 339 animals with intact specimens.

**Table 3:** Distribution of tick species by host species.

Tick Species	Cattle (n)	Sheep (n)	Goat (n)	Total (n)	% (N = 545)
*Dermacentor niveus*	9	0	0	9	1.65
*Dermacentor marginatus*	15	3	9	27	3.48
*Rhipicephalus sanguineus*	9	17	0	26	4.77
*Rhipicephalus turanicus*	41	31	0	72	13.21
*Rhipicephalus bursa*	16	49	0	65	11.92
*Haemaphysalis punctata*	25	9	0	34	6.25
*Haemaphysalis sulcata*	23	18	0	41	7.52
*Hyalomma marginatum*	65	16	7	83	15.22
*Hyalomma a. anatolicum*	85	24	4	112	20.55
*Hyalomma a. excavatum*	58	11	1	70	12.84
*Hyalomma d. detritum*	11	3	0	14	2.56
Total	357	181	21	545	100

Only 7 intact specimens were recovered from goats; the low number precludes species-level statistical comparison for this host group. Goats had the lowest infestation rate (15.2%), significantly lower than the provincial average (*p* = 0.006).

**Table 4:** Seasonal distribution of tick species.

Tick Species	Spring	Summer	Autumn	Winter	Total
*Dermacentor niveus*	9	0	0	0	9
*Dermacentor marginatus*	10	2	7	0	19
*Rhipicephalus sanguineus*	11	15	0	0	26
*Rhipicephalus turanicus*	34	35	3	0	72
*Rhipicephalus bursa*	31	33	1	0	65
*Haemaphysalis punctata*	18	10	6	0	34
*Haemaphysalis sulcata*	19	14	8	0	41
*Hyalomma marginatum*	24	59	0	0	83
*Hyalomma a. anatolicum*	43	65	4	0	112
*Hyalomma a. excavatum*	30	40	0	0	70
*Hyalomma d. detritum*	5	9	0	0	14
**Total**	234	282	29	0	545

Pairwise comparisons: spring vs. autumn (*p* < 0.001), summer vs. autumn (*p* < 0.001). Spring vs. summer (*p* = 0.062, not significant after correction. Bonferroni-adjusted significance level: α = 0.0125.

## Data Availability

All data generated and analyzed during this study are included in the published article.

## Abbreviations

The following abbreviations are used in this manuscript:

CCHF	Crimean-Congo Hemorrhagic Fever
TÜİK	Türkiye Statistical Institute

## References

[1] Keskin A., Selçuk A.Y. (2021). A survey for tick (Acari: Ixodidae) infestation on some wild mammals and the first record of *Ixodes trianguliceps* Birula in Turkey. Syst. Appl. Acarol..

[2] Ahrabi S.Z., Pınarlık F., Akyıldız G., Kuşkucu M., Kar S., Ergönül Ö., Keleş A.G., TEH Vector-Borne Infections Study Group (2025). Human tick biting and tick-borne disease risk in Türkiye: Systematic review. PLoS Negl. Trop. Dis..

[3] René-Martellet M., Minard G., Massot R., Van V.T., Moro C.V., Chabanne L., Mavingui P. (2017). Bacterial microbiota associated with *Rhipicephalus sanguineus* (s.l.) ticks from France, Senegal and Arizona. Parasites Vectors.

[4] Robbins R.G., Nava S., Ronai I., Chong K.L., Guglielmone A.A. (2025). Type specimens of the world’s hard tick species (Acari: Ixodida: Ixodidae): Collection data and depositories for all valid names and the current status of invalid names. Zootaxa.

[5] Riabi H.R.A., Rad M.T., Fazlalipour M., Khakifirouz S., Ahmadi R. (2023). Evaluation of CCHF Infection in Hard Ticks in Razavi Khorasan Province, Iran. J. Res. Health.

[6] Incı A., Yıldırım A., Düzlü Ö., Doğanay M., Aksoy S. (2016). Tick-Borne Diseases in Turkey: A Review Based on One Health Perspective. PLoS Negl. Trop. Dis..

[7] Türkiye İstatistik Kurumu (TÜİK) (2024). Hayvancılık İstatistikleri.

[8] Rooman M., Assad Y., Tabassum S., Sultan S., Ayaz S., Khan M.F., Khan S.N., Rehman A. (2021). A cross-sectional survey of hard ticks and molecular characterization of *Rhipicephalus microplus* parasitizing domestic animals of Khyber Pakhtunkhwa, Pakistan. PLoS ONE.

[9] Ali A., Khan M.A., Zahid H., Yaseen P.M., Khan M.Q., Nawab J., Rehman Z.U., Ateeq M., Khan S., Ibrahim M. (2019). Seasonal Dynamics, Record of Ticks Infesting Humans, Wild and Domestic Animals and Molecular Phylogeny of *Rhipicephalus microplus* in Khyber Pakhtunkhwa Pakistan. Front. Physiol..

[10] Rehman A., Nijhof A.M., Sauter-Louis C., Schauer B., Staubach C., Conraths F.J. (2017). Distribution of ticks infesting ruminants and risk factors associated with high tick prevalence in livestock farms in the semi-arid and arid agro-ecological zones of Pakistan. Parasites Vectors.

[11] Boyard C. (2007). Facteurs Environnementaux de Variation de L’abondance des Tiques *Ixodes ricinus* Dans Des Zones D’étude Modèles en Auvergne. Ph.D. Thesis.

[12] Yılmaz A., Değer M.S. (2011). Determination and seasonal distribution of tick species on cattle and sheep in the Van and Ercis region. Van Vet. Fak. Derg..

[13] Aydın L., Bakırcı S. (2007). Geographical distribution of ticks in Turkey. Parasitol. Res..

[14] Bursali A., Keskin A., Tekin S. (2012). A review of the ticks (Acari: Ixodida) of Turkey: Species diversity, hosts and geographical distribution. Exp. Appl. Acarol..

[15] Estrada-Peña A., Gray J., Kahl O., Lane R.S., Nijhof A.M. (2013). Research on the ecology of ticks and tick-borne pathogens methodological principles and caveats. Front. Cell. Infect. Microbiol..

[16] Guglielmone A.A., Robbins R.G., Apanaskevich D.A., Petney T.N., Estrada-Peña A., Horak I.G., Shao R., Barker S.C. (2010). The Argasidae, Ixodidae and Nuttalliellidae (Acari: Ixodida) of the world: A list of valid species names. Zootaxa.

[17] Bakheit M.A., Latif A.A., Vatansever Z., Seitzer U., Ahmed J. (2012). The huge risks due to *Hyalomma* ticks. Arthropods as Vectors of Emerging Diseases.

[18] Aydın M.F., Dumanlı N. (2019). Tick-borne pathogens in small ruminants in Turkey: A systematic review. Turk. Vet. J..

[19] Telmadarraiy Z., Kooshki H., Edalat H., Vatandoost H., Bakhshi H., Faghihi F., Hosseini-Chegeni A., Oshaghi M.A. (2022). Study on Hard and Soft Ticks of Domestic and Wild Animals in Western Iran. J. Arthropod-Borne Dis..

[20] Incı A., Yıldırım A., Düzlü Ö. (2016). The Current Status of Ticks in Turkey: A 100-Year Period Review from 1916 to 2016. Turk. Parazitol. Derg..

[21] Sonenshine D.E., Roe R.M. (2013). Biology of Ticks, Volume 2.

[22] Kar S., Keles A.G. (2021). Possible direct and human-mediated impact of climate change on tick populations in Turkey. Climate, Ticks and Disease.

[23] Bursali A., Tekin S., Orhan M., Keskin A., Ozkan M. (2010). Ixodid ticks (Acari: Ixodidae) infesting humans in Tokat Province of Turkey: Species diversity and seasonal activity. J. Vector Ecol..

